# Hypertensive disorders of pregnancy and neonatal outcomes in twin vs. singleton pregnancies after assisted reproductive technology

**DOI:** 10.3389/fped.2022.839882

**Published:** 2022-09-02

**Authors:** Ting Liu, Rui Gao, Yong Liu, Ke Zhao, Xiaolin Su, Hin Ching Wong, Luyao Li, Binbin Xie, Yuanyan Huang, Chuhui Qiu, Jiang He, Chaoqun Liu

**Affiliations:** ^1^Department of Nutrition, School of Medicine, Jinan University, Guangzhou, China; ^2^Shenzhen Birth Cohort Study Center, Nanshan Maternity and Child Healthcare Hospital of Shenzhen, Shenzhen, China; ^3^Department of Laboratory Medicine, Hospital of Stomatology, Anhui Medical University, Hefei, China; ^4^Department of Mathematics and Physics, School of Biomedical Engineering, Southern Medical University, Guangzhou, China

**Keywords:** hypertensive disorders of pregnancy, assisted reproductive technology, adverse neonatal outcomes, singleton, twin

## Abstract

**Objective:**

Hypertensive disorders of pregnancy (HDP) are associated with an increased risk of adverse neonatal outcomes. Although twin pregnancies had a higher risk of developing HDP, it is not known whether HDP in twins will increase the risk of adverse neonatal outcomes. We aimed to assess whether this association differed in singleton and twin pregnancies in women who conceived with assisted reproductive technology (ART).

**Methods:**

We finally included 193,590 live births born *via* ART from the National Vital Statistics System (NVSS) for the years 2015–2019. We used Log-binomial regression to evaluate the associations between HDP and the risk of adverse neonatal outcomes in ART mothers.

**Results:**

Among 193,590 ART-treated mothers, there were 140,870 and 52,720 mothers who had singleton pregnancies and twin pregnancies, respectively. Those ART mothers with twin pregnancies had a higher rate of HDP than singleton pregnancies (20.5% vs. 11.0%). In singleton pregnancies, the risks of preterm birth [adjusted risk ratio (aRR)): 2.80, 95% CI 2.67–2.93], low birth weight (aRR: 2.80, 95% CI 2.67–2.93), small for gestational age (aRR: 1.41, 95% CI 1.34–1.49), 5 min Apgar <7 (aRR: 1.66, 95% CI 1.50–1.83) and cesarean section (aRR: 1.23, 95% CI 1.21–1.25) were significantly higher in HDP mothers than in non-HDP mothers respectively. However, in contrast to singleton pregnancies, these associations were weak or reversed in twin pregnancies, after adjusting for confounding factors.

**Conclusion:**

In ART-treated women, although twin pregnancies had a higher HDP rate, the risk of adverse neonatal outcomes associated with HDP was lower than that of singletons.

## Introduction

Assisted reproductive technology (ART) becomes a popular treatment that is widely used for infertile couples in the past decades (1) and proved effective in achieving considerable rates of successful artificial conception (2). The use of ART has risen steadily in the United States largely due to childbearing at later ages becoming more common (3–5). In the United States between 2012 and 2014, there were 191,250 babies born from ART, which accounted for 1.6% of all births, and the number has more than doubled over the past decade (6). In addition, ART constitutes a major risk factor for the genesis of twin and higher-order multiple births (7).

Hypertensive disorders of pregnancy (HDP) are one of the leading causes of adverse maternal and neonatal outcomes during pregnancy, including gestational hypertension (GH) and preeclampsia/eclampsia (8), which occurs 2–3 times more frequently in multiple pregnancies compared with singleton pregnancies (9). It has been established that HDP predisposes infants to adverse neonatal outcomes in singleton pregnancies, such as preterm birth (PTB), low birth weight (LBW), small for gestational age (SGA), and so on (10–12). However, data regarding the association of HDP with adverse neonatal outcomes in twin pregnancies are conflicting. While some researchers have reported that HDP does not have a severe effect on twin births (13), others have reported the contrary (14). Nonetheless, most of these findings are based on spontaneously conceived pregnancies which may not be applicable to ART pregnancies, especially for twin pregnancies, due to their different demographic and clinical factors, including advanced maternal age, and high prepregnancy body mass index (BMI), primiparity, and preexisting diabetes. There is the concern that twin pregnancies have a higher risk of developing HDP, however, it is presently not known whether HDP in ART twin pregnancies will increase the risk of adverse neonatal outcomes.

Thus, there is an urgent need for a large, diverse population database with substantial available information for potential confounding factors to establish this. The aim of this study was to investigate the potentially differential effect of HDP on adverse neonatal outcomes in ART twin pregnancies compared with ART singleton pregnancies.

## Materials and methods

### Study population and data sources

This study utilizes data from the National Vital Statistics System (NVSS), an official program that provides an extensive and longitudinal vital statistic database. The NVSS natality data file includes all births occurring within the United States in 50 states and the District of Columbia in the U.S which are based on information for all births registered in the reporting areas. NVSS uses two worksheets to better collect data, including the mother's worksheet and the facility worksheet for all live births in the 50 states and the District of Columbia. The information about maternal demographic characteristics is directly obtained from the mother using the mother's worksheet. Medical and health information of the mother and infant is extracted from the facilities worksheet completed by hospital staff. For analysis, data including all ART mothers who had live births and available information for maternal hypertension and birth outcomes from the years 2015 to 2019 were selected from NVSS. Women with any of the following conditions were excluded from the analyses: maternal age <18 years; chronic hypertension before pregnancy; gestational age at birth <22 weeks or > 42 weeks; triplets or higher plurality; births with missing data for any of the following variables: gestational age at birth, birth weight, parity, 5-min Apgar, neonatal death. This present study followed the Strengthening the Reporting of Observational Studies in Epidemiology Guidelines (15). Because these records are publicly available and the data are de-identified, Institutional Review Board approval was not required for this study.

### Study factors

In the current study, ART procedures referred to how both egg and sperm are handled in the laboratory (e.g., *in vitro* fertilization (IVF), Intracytoplasmic sperm injection (ICSI), gamete intrafallopian transfer (GIFT), Zygote Intrafallopian Transfer (ZIFT)). However, the type of ART treatment was not specified in the study. Maternal age was divided into the following categories: < 30, 30–34, 35–39, and 40 years or older. Maternal race/ethnicity was classified as Hispanic, non-Hispanic white, non-Hispanic black, and others. Maternal education levels were categorized as lower than college, college, higher than college, and unknown. Marital status was categorized as married, unmarried, and unknown. Prepregnancy body mass index (BMI) was classified into the following categories: underweight (< 18.5 kg/m^2^), normal (18.5–24.9 kg/m^2^), overweight (25.0–29.9 kg/m^2^), obesity I (30.0–34.9 kg/m^2^), obesity class II (35.0–39.9 kg/m^2^), and obesity class III (≥ 40.0 kg/m^2^). Timing of initiation of prenatal care was categorized based on the trimester of the first prenatal visit as no prenatal care, 1st−3rd month, 4th−6th month, 7th–final month, and unknown. Parity was divided into two groups: primiparous and multiparous. Smoking before and during pregnancy, and prepregnancy diabetes were coded as “yes,” “no,” and “missing.” The infant sex was classified as male and female. Plurality was divided into two groups: singleton and twin pregnancies.

### Neonatal outcomes

Information on neonatal outcomes was extracted from the facilities worksheet. PTB was defined as birth occurring before 37 completed weeks of gestation based on the obstetric estimate of gestation at delivery, consistent with the classification of International Statistical Classification of Diseases, Ninth Revision (ICD−9) and the International Statistical Classification of Diseases, Tenth Revision (ICD−10) definitions. LBW was defined as birth weight < 2,500 g. SGA was defined as neonatal birth weight < 10th percentile for gestational age, according to the USA sex-specific reference (16). The other neonatal outcomes include neonatal death of infants aged 0–27 days and Apgar score < 7 at 5 min.

### Hypertensive disorders of pregnancy

In our study, the key explanatory variable of interest was HDP which includes gestational hypertension (GH), preeclampsia, and eclampsia. In the United States during the studied period (8), gestational hypertension is defined as a systolic blood pressure of 140 mmHg or more or diastolic blood pressure of 90 mmHg or more, or both, on two occasions at least 4 h apart after 20 weeks of gestation, in a woman with previously normal blood pressure. Preeclampsia is a disorder of pregnancy presenting as new-onset hypertension, which occurs most often after 20 weeks of gestation and frequently near term. Eclampsia is defined as hypertension with proteinuria with generalized seizures or coma, that may include pathologic edema. However, the revised birth certificate did not allow the distinction between gestational hypertension and preeclampsia, including *de novo* hypertension with proteinuria (17). As a result, we could not study preeclampsia separately.

### Statistical analysis

Baseline characteristics and neonatal outcomes presented as numbers or proportions were compared between ART women with and without HDP for the singleton, and twin births separately. All independent variables were transformed into categorical variables. The analysis of the associations of HDP with neonatal outcomes was performed separately for each of the two comparison groups. Log-binomial regression was used to calculate adjusted risk ratios (aRRs) and 95% confidence intervals (CIs) using no HDP group as the reference group. To reduce confounding effects, we adjusted for several potential confounders including the mother's age, race/ethnicity, education levels, marital status, parity, the timing of initiation of prenatal care, prepregnancy BMI, infant sex, and prepregnancy diabetes. For all analyses, two-sided *P*-values were considered statistically significant if < 0.05. Statistical analyses were implemented in R statistical software (R version 3.6.3).

## Results

### Study characteristics

**Figure 1:**
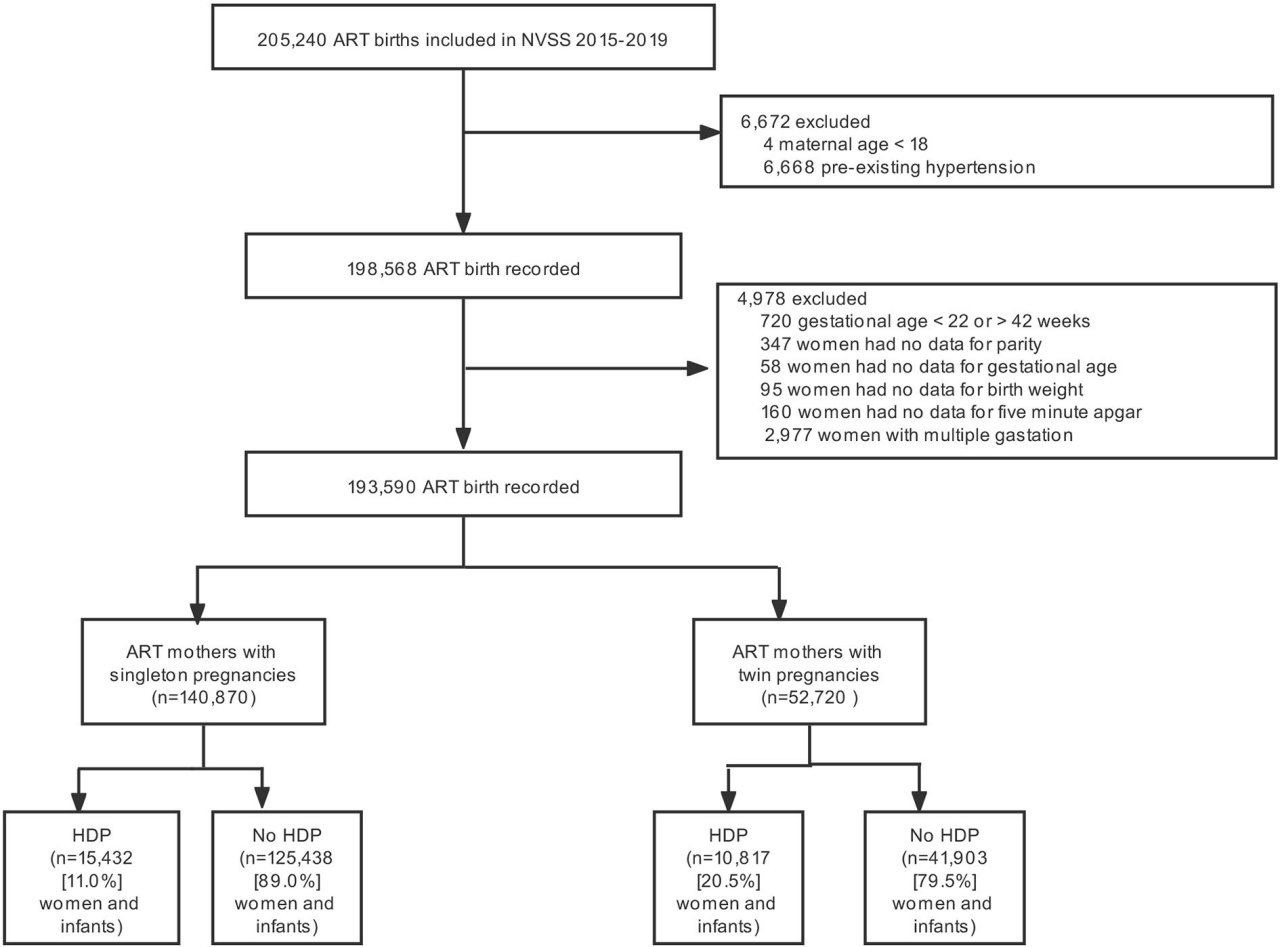
Participant flow chart. NVSS, US National Vital Statistics System; ART, Assisted reproductive technology; HDP, Hypertensive disorders of pregnancy.

The study included a total of 205,240 ART-treated live birth records in the U.S. from 2015 to 2019. Of the 193,590 ART mothers and infants who met inclusion criteria, 140,870 had a singleton pregnancy, and 52,720 had a twin pregnancy (Figure 1). The incidence of HDP according to different plurality was 11.0% for singletons and 20.5% for twins, respectively.

The characteristics of study subjects stratified by HDP status in the singleton and twin pregnancies groups are shown in Table 1. Women who gave birth *via* ART were generally older, with an average age of over 34 years old. Compared with women without HDP, women with HDP tend to be older, more likely to be overweight and obese, non-Hispanic white, unmarried, and more likely to have lower parity and prepregnancy diabetes in both singleton and twin groups (*P* < 0.05). Compared with women who had no HDP, women with HDP tend to have less education and smoking before pregnancy otherwise only found in singleton groups (*P* < 0.05). In addition, there were no statistically significant differences between the groups on the timing of initiation of prenatal care, maternal smoking during pregnancy, and infant sex (*P* > 0.05).

**Table 1:** Demographic characteristics according to HDP in ART mothers–NVSS, 2015–2019.

**Characteristic**	**Singletons**			**Twins**		
	**No HDP**	**HDP**	***P***	**No HDP**	**HDP**	***P***
Total population, *n*	125,438	15,432		41,903	10,817	
Age, years, mean (SD)	35.7 (5.08)	36.1 (5.42)	< 0.001	35.0 (5.14)	35.6 (5.46)	< 0.001
Age, years, *n (%)*			< 0.001			< 0.001
< 30	12,536 (10.0)	1562 (10.1)		5,420 (12.9)	1309 (12.1)	
30–34	40,935 (32.6)	4855 (31.5)		14,861 (35.5)	3,589(33.2)	
35–39	44,754 (35.7)	5,086 (33.0)		14,150 (33.8)	3,502 (32.4)	
≥ 40	27,213 (21.7)	3929 (25.5)		7,472 (17.8)	2,417 (22.3)	
Race/ethnicity, *n (%)*			< 0.001			< 0.001
Hispanic	10,217 (8.1)	1,238 (8.0)		3,969 (1.4)	1,018 (9.4)	
Non–Hispanic white	86,581 (69.0)	11,198 (72.6)		27,958 (66.7)	7,749 (71.6)	
Non–Hispanic black	5,463 (4.4)	842(5.5)		2,145 (5.1)	553(5.1)	
Other	19,400 (15.5)	1,788 (11.6)		6,206 (14.8)	1,184 (10.9)	
Unknown	3,777 (3.0)	266 (2.4)		1,625 (3.9)	313 (2.9)	
Education levels, *n (%)*			< 0.001			0.319
Lower than college	27,282 (21.7)	4,068 (26.4)		11,743 (28.0)	3,145 (29.1)	
College	45,270 (36.1)	5,476 (35.5)		14,829 (35.4)	3,825 (35.4)	
Higher than college	49,047 (39.1)	5,506 (35.7)		13,497 (32.2)	3,492 (32.3)	
Unknown	3,839 (3.1)	382 (2.5)		54 (2.5)	18 (2.3)	
Marital status, *n (%)*			< 0.001			0.027
Married	104,371 (83.2)	12,855 (83.3)		35,322 (84.3)	9,184 (84.9)	
Unmarried	8,387 (6.7)	1,224 (7.9)		2,823 (6.7)	750 (6.9)	
Unknown	12,680 (10.1)	1,353 (8.8)		3,758 (9.0)	883 (8.2)	
Parity, *n (%)*			< 0.001			< 0.001
Primiparous	74,381 (59.3)	10,907 (70.7)		14,127 (33.7)	4,165 (38.4)	
Multiparous	51,057 (40.7)	4,525 (29.3)		27,776 (66.3)	6,661 (61.6)	
Smoking before pregnancy, *n (%)*			< 0.001			0.151
Yes	1,284 (1.0)	221 (1.4)		513 (1.2)	114 (1.1)	
No	123,831 (98.7)	15,173 (98.3)		41,260 (98.5)	10,681 (98.7)	
Unknown	323 (0.3)	38 (0.2)		130 (0.3)	22 (0.2)	
Smoking during pregnancy, *n (%)*			0.032			0.028
Yes	517 (0.4)	82 (0.5)		188 (0.4)	32 (0.3)	
No	124,593 (99.3)	15,315 (99.2)		41,583 (99.2)	10,762 (99.5)	
Unknown	328 (0.3)	35 (0.2)		132 (0.3)	23 (0.2)	
Timing of initiation of prenatal care, *n (%)*			0.002			0.220
1st−3rd month	110,538 (88.1)	13,601 (88.1)		36,310 (86.7)	9,363 (86.6)	
4th−6th month	10,710 (8.5)	1,305 (8.5)		3,927 (9.4)	978 (9.0)	
7th–final month	1,846 (1.5)	176 (1.1)		577 (1.4)	145 (1.3)	
No prenatal care	270 (0.2)	46 (0.3)		117 (0.3)	19 (0.2)	
Unknown	2,074 (1.7)	304 (2.0)		972 (2.3)	312 (2.9)	
Prepregnancy BMI, kg/m2, *n (%)*			<0.001			<0.001
Underweight: 18.5	3,197 (2.5)	178 (1.2)		1,020 (2.4)	148 (1.4)	
Normal: 18.5–24.9	66,389 (52.9)	5,268 (34.1)		20,823 (49.7)	4,295 (39.7)	
Overweight: 25.0–29.9	31,602 (25.2)	4,350 (28.2)		10,921 (26.1)	3,063 (28.3)	
Obesity I: 30.0–34.9	13,774 (11.0)	2,843 (18.4)		5,164 (12.3)	1,707 (15.8)	
Obesity II: 35.0–39.9	5,701 (4.5)	1,511 (9.8)		2,049 (4.9)	914 (8.4)	
Obesity III: ≥ 40.0	2,714 (2.2)	976 (6.3)		997 (2.4)	486 (4.5)	
Unknown	2,061 (1.6)	306 (2.0)		929 (2.2)	204 (1.9)	
Prepregnancy diabetes, *n (%)*			<0.001			<0.001
Yes	964 (0.8)	282 (1.8)		267 (0.6)	140 (1.3)	
No	124,474 (99.2)	15,150 (98.2)		41,636 (99.4)	10,677 (98.7)	
Infant sex, *n (%)*			0.973			0.029
Male	64,073 (51.1)	7,885 (51.1)		21,521 (51.4)	5,428 (50.2)	
Female	61,365 (48.9)	7,547 (48.9)		20,382 (48.6)	5,389 (49.8)	

HDP, hypertensive disorders of pregnancy (gestational hypertension or preeclampsia/eclampsia).

### Associations between HDP and adverse neonatal outcomes in singleton and twin gestation of ART mothers

The risk of adverse neonatal outcomes in ART mothers with and without HDP by plurality was shown in Table 2. As in the singleton births, the cumulative rates of PTB (9.4% vs. 24.5%), LBW (6.5% vs. 19.5%), SGA (9.6% vs. 12.7%) and 5-min Apgar < 7 (2.0% vs. 3.6%) in HDP women were significantly higher than in non HDP women (*P* < 0.05). Furthermore, logistic regression analyses showed that the risk of PTB (aRR: 2.62, 95% CI 2.53–2.71), LBW (aRR: 3.27, 95% CI 3.12–3.44), SGA (aRR: 1.41, 95% CI 1.34–1.49) and 5-min Apgar < 7 (aRR: 1.66, 95% CI 1.50–1.83), cesarean section (aRR: 1.23, 95% CI 1.21–1.25) in singleton births were significantly higher in HDP mothers than in non-HDP mothers respectively, after controlling for maternal age, race/ethnicity, maternal education levels, marital status, parity, the timing of initiation of prenatal care, prepregnancy BMI, infant sex, and prepregnancy diabetes. As expected, the absolute rates of these adverse neonatal outcomes were significantly higher in twin pregnancies compared with singleton pregnancies, irrespective of HDP status. However, in contrast to singleton pregnancies, these associations were weak or reversed in twin pregnancies, after adjusting for confounding factors [aRR of PTB (1.42, 95% CI 1.39–1.44), LBW (1.25, 95% CI 1.22–1.28), SGA (0.88, 95% CI 0.84–0.92), 5-min Apgar < 7 (0.93, 95% CI 0.85–1.02), neonatal death (0.26, 95% CI 0.21–0.33)] and cesarean section (aRR: 1.07, 95% CI 1.04–1.11).

**Table 2:** Association between HDP and adverse neonatal outcomes by plurality in ART mothers.

**Adverse neonatal outcomes**	**Singletons**				**Twins**			
	**No HDP**	**HDP**	**Crude RR (95% CI)**	**Adjusted RR ^b^** **(95% CI)**	**No HDP**	**HDP**	**Crude RR (95% CI)**	**Adjusted RR ^b^** **(95% CI)**
Preterm birth (< 37 wk)	11,732 (9.4)	3,777 (24.5)	2.62 (2.53–2.70)	2.62 (2.53–2.71)	22,614 (54.0)	8,200 (75.8)	1.41 (1.39–1.42)	1.42 (1.39–1.44)
Low birth weight (< 2500 g)	8,118 (6.5)	3012 (19.5)	3.02 (2.89–3.15)	3.00 (2.87–3.13)	20,791 (49.6)	6659 (61.6)	1.24 (1.22–1.26)	1.25 (1.22–1.28)
Small for gestational age ^a^	12,055 (9.6)	1,965 (12.7)	1.31 (1.25–1.38)	1.30 (1.24–1.37)	9,517 (22.7)	2,175 (20.1)	0.89 (0.85–0.92)	0.88 (0.84–0.92)
Five–minute Apgar < 7	2,483 (2.0)	553 (3.6)	1.81 (1.65–1.98)	1.66 (1.50–1.83)	1,872 (4.5)	459 (4.2)	0.95 (0.86–1.05)	0.93 (0.85–1.02)
Neonatal death	220 (0.2)	34 (0.2)	1.26 (0.88–1.80)	1.01 (0.67–1.52)	403 (1.0)	36 (0.3)	0.35 (0.25–0.49)	0.26 (0.21–0.33)
Cesarean section	57,850 (46.1)	8,787 (56.9)	1.24 (1.22–1.25)	1.23 (1.21–1.25)	32,860 (78.4)	9,263 (85.6)	1.08 (1.04–1.11)	1.07 (1.04–1.11)

Data are presented as n (percentage), or relative risk (95% confidence interval).

HDP, hypertension in pregnancy (gestational hypertension or preeclampsia/eclampsia); RR, relative risk.

a Based on the 2017 US birthweight reference using Obstetric Estimates of Gestation of Aris et al. (16).

b Adjusted for maternal age, race/ethnicity, maternal education levels, marital status, parity, timing of initiation of prenatal care, prepregnancy BMI, infant sex and prepregnancy diabetes.

**Figure 2:**
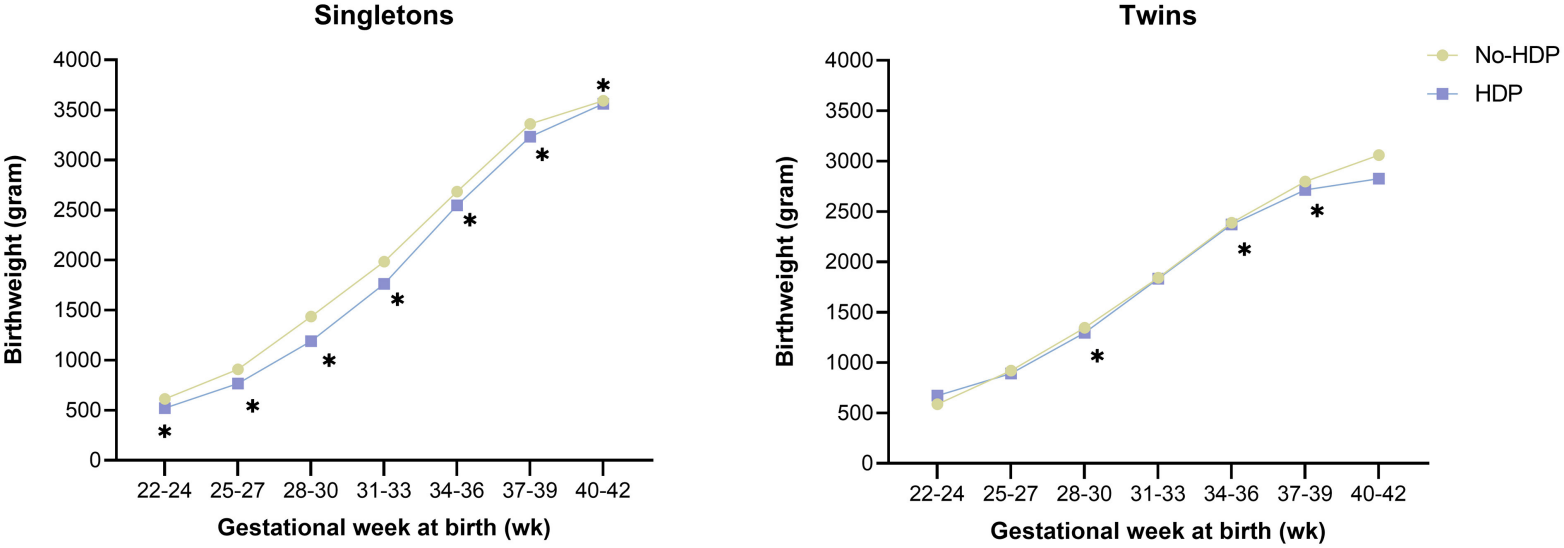
Relationship between HDP and gestational age-specific birthweight by plurality among ART mothers. HDP, Hypertensive disorders in pregnancy. Asterisk indicates a value of *P* < 0.05.

To reduce the effect of gestational age on birth weight, we calculate gestational age-specific birthweight (Figure 2). In the singleton group, the average gestational age-specific birthweight was lower in women with HDP compared with women without HDP. However, these differences were minor in the twins.

Since the association between HDP and adverse neonatal outcomes differed significantly by maternal age (*P*_interaction_ < 0.001), stratified analysis by maternal age is needed. In the singleton group, HDP significantly increased the risk of PTB, LBW, SGA, 5-min Apgar < 7, and cesarean section among those aged < 30 years, 30–34 years, 35–39 years, and ≥ 40 years (Table 3). In twin pregnancies, the corresponding aRR of PTB, LBW, SGA, 5-min Apgar < 7, and cesarean section were much lower or showed reversed associations among those aged <30 years, 30–34 years, 35–39 years, and ≥ 40 years. In contrast, the risk of neonatal death even showed a protective effect in twin pregnancies. The association was also generally consistent across subgroups by maternal prepregnancy obesity status (Table 4).

**Table 3:** Stratified analysis for the association of HDP and adverse neonatal outcomes in ART mothers by age group.

**Adverse neonatal outcome**	**Singletons**				**Twins**			
	**Crude RR (95% CI)**	***P***	**Adjusted RR ^b^ (95% CI)**	***P***	**Crude RR (95% CI)**	***P***	**Adjusted RR ^b^ (95% CI)**	***P***
**Preterm birth (< 37 wk)**								
< 30	2.36 (2.13–2.62)	< 0.001	2.34 (2.10–2.60)	< 0.001	1.27 (1.23–1.32)	< 0.001	1.29 (1.24–1.34)	< 0.001
30–34	2.54 (2.39–2.70)	< 0.001	2.52 (2.37–2.69)	< 0.001	1.37 (1.34–1.41)	< 0.001	1.39 (1.35–1.42)	< 0.001
35–39	2.67 (2.52–2.83)	< 0.001	2.70 (2.54–2.86)	< 0.001	1.46 (1.42–1.49)	< 0.001	1.46 (1.42–1.50)	< 0.001
≥ 40	2.67 (2.51–2.84)	< 0.001	2.66 (2.50–2.84)	< 0.001	1.47 (1.43–−1.51)	< 0.001	1.49 (1.44–1.54)	< 0.001
**Low birth weight (< 2500 g)**								
< 30	2.40 (2.12–2.72)	< 0.001	2.36 (2.08–2.69)	< 0.001	1.11 (1.06–1.17)	< 0.001	1.14 (1.08–1.20)	< 0.001
30–34	2.92 (2.72–3.13)	< 0.001	2.91 (2.71–3.13)	< 0.001	1.22 (1.19–1.26)	< 0.001	1.23 (1.20–1.27)	< 0.001
35–39	3.15 (2.94–3.37)	< 0.001	3.17 (2.95–3.39)	< 0.001	1.25 (1.21–1.29)	< 0.001	1.25 (1.20–1.29)	< 0.001
≥ 40	3.16 (2.94–3.40)	< 0.001	3.10 (2.87–3.35)	< 0.001	1.34 (1.30–1.40)	< 0.001	1.36 (1.30–1.41)	< 0.001
**Small for gestational age** ^a^								
< 30	1.24 (1.05–−1.46)	0.011	1.20 (1.04–1.40)	0.014	0.94 (0.83–1.06)	0.298	0.94 (0.83–1.07)	0.331
30–34	1.41 (1.29–1.54)	< 0.001	1.35 (1.25–1.46)	< 0.001	0.90 (0.84–0.97)	0.004	0.90 (0.84–0.97)	0.005
35–39	1.38 (1.28–1.48)	< 0.001	1.37 (1.27–1.48)	< 0.001	0.89 (0.83–0.95)	0.001	0.89 (0.83–0.96)	0.001
≥ 40	1.27 (1.14–1.41)	< 0.001	1.26 (1.08–1.32)	< 0.001	0.84 (0.77–0.92)	< 0.001	0.80 (0.72–0.88)	< 0.001
**Five–minute Apgar < 7**								
< 30	1.58 (1.21–2.06)	0.001	1.62 (1.23–2.14)	0.001	0.60 (0.46–0.80)	< 0.001	0.62 (0.46–0.83)	< 0.001
30–34	1.68 (1.43–1.97)	< 0.001	1.72 (1.46–2.03)	< 0.001	0.96 (0.81–1.14)	0.627	0.94 (0.79–1.13)	0.523
35–39	1.97 (1.69–2.31)	< 0.001	1.76 (1.48–2.10)	< 0.001	1.12 (0.95–1.34)	0.186	1.05 (0.87–1.26)	0.608
≥ 40	1.94 (1.61–2.34)	< 0.001	1.78 (1.45–2.19)	< 0.001	1.08 (0.85–1.37)	0.540	1.13 (0.88–1.45)	0.347
**Neonatal death**								
< 30	0.89 (0.32–2.50)	0.828	1.08 (0.38–3.50)	0.890	0.18 (0.07–0.49)	0.001	0.20 (0.07–0.55)	0.002
30–34	1.11 (0.57–2.14)	0.757	1.11 (0.53–2.31)	0.787	0.32 (0.18–0.60)	0.323	0.20 (0.09–0.46)	< 0.001
35–39	1.42 (0.75–2.69)	0.277	1.46 (0.75–2.85)	0.266	0.62 (0.38–1.02)	< 0.001	0.49 (0.26–0.91)	0.023
≥ 40	1.56 (0.76–3.21)	0.229	1.36 (0.57–3.27)	0.490	0.18 (0.06–0.56)	0.175	0.15 (0.04–0.61)	0.008
**Cesarean section**								
< 30	1.33 (1.25–1.41)	< 0.001	1.34 (1.26–1.42)	< 0.001	1.14 (1.11–1.18)	< 0.001	1.14 (1.11–1.18)	< 0.001
30–34	1.26 (1.22–1,29)	< 0.001	1.25 (1.21–1.29)	< 0.001	1.09 (1.08–1.11)	< 0.001	1.09 (1.08–1.11)	< 0.001
35–39	1.21 (1.18–1.25)	< 0.001	1.21 (1.17–1.24)	< 0.001	1.07 (1.06–1.09)	< 0.001	1.07 (1.06–1.09)	< 0.001
≥ 40	1.18 (1.15–1.20)	< 0.001	1.17 (1.14–1.19)	< 0.001	–	–	–	–

Data are presented as n(percentage), or relative risk (95% confidence interval).

HDP, hypertension in pregnancy (gestational hypertension or preeclampsia/eclampsia); RR, relative risk.

a Based on the 2017 US birthweight reference using Obstetric Estimates of Gestation of Aris et al. (16).

b Adjusted for race/ethnicity, maternal education levels, marital status, parity, timing of initiation of prenatal care, prepregnancy BMI, infant sex and prepregnancy diabetes.

**Table 4:** Stratified analysis for the association of HDP and adverse neonatal outcomes in ART mothers by Prepregnancy BMI.

**Adverse neonatal outcome**	**Singletons**				**Twins**			
	**Crude RR (95% CI)**	***P***	**Adjusted RR ^b^ (95% CI)**	***P***	**Crude RR (95% CI)**	***P***	**Adjusted RR ^b^ (95% CI)**	***P***
**Preterm birth (< 37 wk)**								
Underweight: 18.5	2.94 (2.24–3.86)	< 0.001	2.97 (2.24–3.93)	< 0.001	1.37 (1.25–1.51)	< 0.001	1.38 (1.25–1.53)	< 0.001
Normal: 18.5–24.9	2.98 (2.82–3.15)	<0.001	2.98 (2.81–3.15)	<0.001	1.44 (1.41–1.47)	<0.001	1.45 (1.42–1.48)	<0.001
Overweight: 25.0–29.9	2.38 (2.24–2.54)	<0.001	2.34 (2.19–2.50)	<0.001	1.38 (1.34–1.42)	<0.001	1.39 (1.35–1.43)	<0.001
Obesity: ≥ 30.0	2.09 (1.97–2.12)	<0.001	2.10 (1.98–2.24)	<0.001	1.37 (1.34–1.41)	<0.001	1.38 (1.34–1.41)	<0.001
**Low birth weight (< 2500 g)**								
Underweight: 18.5	3.50 (2.72–4.51)	<0.001	3.38 (2.59–4.40)	<0.001	1.31 (1.18–1.45)	<0.001	1.32 (1.18–1.47)	<0.001
Normal: 18.5–24.9	3.60 (3.39–3.83)	<0.001	3.60 (3.38–3.83)	<0.001	1.30 (1.26–1.33)	<0.001	1.30 (1.27–1.34)	<0.001
Overweight: 25.0–29.9	2.90 (2.69–3.13)	<0.001	2.86 (2.65–3.10)	<0.001	1.25 (1.21–1.30)	<0.001	1.26 (1.21–1.30)	<0.001
Obesity: ≥ 30.0	2.29 (2.13–2.46)	<0.001	2.28 (2.12–2.46)	<0.001	1.20 (1.15–1.24)	<0.001	1.19 (1.14–1.23)	<0.001
**Small for gestational age** ^a^								
Underweight: 18.5	1.32 (1.01–1.74)	0.046	1.29 (0.97–1.72)	0.076	0.86 (0.64–1.14)	0.284	0.84 (0.62–1.14)	0.267
Normal: 18.5–24.9	1.42 (1.33–1.52)	<0.001	1.43 (1.34–1.53)	<0.001	0.91 (0.86–0.97)	0.003	0.91 (0.86–0.97)	0.004
Overweight: 25.0–29.9	1.46 (1.34–1.59)	<0.001	1.42 (1.30–1.55)	<0.001	0.94 (0.87–1.02)	0.134	0.92 (0.84–0.99)	0.035
Obesity: ≥ 30.0	1.31 (1.19–1.43)	<0.001	1.29 (1.17–1.42)	<0.001	0.91 (0.83–0.99)	0.037	0.90 (0.82–0.99)	0.026
**Five–minute Apgar < 7**								
Underweight: 18.5	1.99 (0.93–4.29)	0.078	2.21 (1.03–4.77)	0.043	1.06 (0.38–3.00)	0.912	0.94 (0.29–3.09)	0.917
Normal: 18.5–24.9	2.03 (1.74–2.36)	<0.001	2.08 (1.78–2.43)	<0.001	1.08 (0.92–1.27)	0.332	1.07 (0.91–1.26)	0.415
Overweight: 25.0–29.9	1.53 (1.26–1.84)	<0.001	1.57 (1.30–1.90)	<0.001	0.88 (0.73–1.07)	0.204	0.93 (0.76–1.13)	0.468
Obesity: ≥ 30.0	1.50 (1.29–1.75)	<0.001	1.53 (1.31–1.79)	<0.001	0.71 (0.59–0.86)	<0.001	0.69 (0.56–0.84)	<0.001
**Neonatal death**								
Underweight: 18.5	N/A	N/A	N/A	N/A	3.45 (0.64–18.65)	0.151	3.44 (0.31–37.70)	0.312
Normal: 18.5–24.9	2.07 (1.13–3.81)	0.019	2.07 (1.09–3.91)	0.026	0.42 (0.23–0.76)	0.004	0.33 (0.16–0.67)	0.002
Overweight: 25.0–29.9	0.45 (0.14–1.46)	0.184	0.53 (0.16–1.70)	0.281	0.09 (0.03–0.29)	<0.001	0.07 (0.02–0.29)	<0.001
Obesity: ≥ 30.0	0.80 (0.46–1.39)	0.430	0.83 (0.47–1.46)	0.511	0.25 (0.14–0.45)	<0.001	0.27 (0.15–0.49)	<0.001
**Cesarean section**								
Underweight: 18.5	1.29 (1.10–1.52)	0.001	1.30 (1.11–1.53)	0.002	N/A	N/A	N/A	N/A
Normal: 18.5–24.9	1.21 (1.17–1.24)	<0.001	1.20 (1.16–1.23)	<0.001	1.11 (1.09–1.13)	<0.001	1.10 (1.09–1.12)	<0.001
Overweight: 25.0–29.9	1.17 (1.13–1.20)	<0.001	1.16 (1.13–1.20)	<0.001	1.07 (1.05–1.09)	<0.001	1.07 (1.05–1.09)	<0.001
Obesity: ≥ 30.0	1.15 (1.12–1.18)	<0.001	1.15 (1.12–1.18)	<0.001	N/A	N/A	N/A	N/A

Data are presented as n(percentage), or relative risk (95% confidence interval).

HDP, hypertension in pregnancy (gestational hypertension or preeclampsia/eclampsia); RR, relative risk.

a Based on the 2017 US birthweight reference using Obstetric Estimates of Gestation of Aris et al. (16).

b Adjusted for maternal age, race/ethnicity, maternal education levels, marital status, parity, timing of initiation of prenatal care, infant sex and prepregnancy diabetes.

## Discussion

In this large observational study using the 2015–2019 NVSS live births databases, we observed that in ART-treated mothers, the incidence of HDP was statistically significantly higher in twins than the singleton pregnancies (20.5% vs. 11.0%). In singleton pregnancies, HDP significantly increased the risk of PTB, LBW, SGA, 5-min Apgar < 7, and cesarean section. Compared to singleton pregnancies, the absolute rates of these adverse neonatal outcomes were significantly higher in twin pregnancies, irrespective of HDP status, whereas the risk of adverse neonatal outcomes associated with HDP in twin pregnancies was lower than that in singleton pregnancies.

Generally, HDP affects 5 to 10% of all pregnancies worldwide (18). However, women who conceived by ART are known to have a higher prevalence of overall HDP compared with spontaneous conception (SC). The reason behind this is complex and remains unclear. The CoNARTaS study from Sweden, Denmark, and Norway during the years 1988 to 2007 showed that HDP occurred in 5.9% of ART and 4.7% of SC singleton pregnancies, and in 12.6% of ART and 10.4% of SC twin pregnancies (19). Whether singleton or twin pregnancies, a moderate increase in the risk of HDP was seen in ART compared with SC, while the proportion of HDP was significantly higher in twin pregnancies than in singleton pregnancies, which suggested that ART itself has only a slight effect on the development of HDP, multiple pregnancies might be the primary reason instead. Another study from Australia during the years 2007 to 2011 reported that the rate of GH/PE was higher in ART mothers with twin pregnancies (12.4%) than that in singletons (5.7%) (20). In contrast, we observed that in this American ART population, the incidence of HDP in singleton (11.0%) and twin pregnancies (20.5%) were significantly higher than that of other countries. This disparity may be related to the rapid increase in the incidence of HDP in American women in recent years (21). Recent works indicated that the blood pressure levels during twin pregnancies were higher than those during singleton pregnancies (22). Several possible reasons may explain this finding. In twin pregnancies, maternal gestational weight gain more and faster than the singletons, in turn, parallel increases cardiac output in mothers (23). At present, the diagnosis of HDP is mainly based on blood pressure, and the classification criteria for singleton and twin pregnancies are the same, which can directly increase the absolute rate of HDP in twin pregnancies. Numerous research demonstrated that twin pregnancies can cause angiogenic imbalances, which are linked to an increased placental mass, leading to increased circulating levels of sFlt1 and sFLT1/PlGF ratios, thereby increasing the risk of preeclampsia (24–26). The thesis may explain the elevated risks of HDP in twin pregnancies.

Numerous studies indicated that HDP was associated with an increased risk of adverse neonatal outcomes, especially for adverse birth outcomes in singleton pregnancies (27–29). In this study, we also found that ART mothers with HDP tended to have a higher risk of PTB, LBW, SGA, and 5-min Apgar < 7 than those without HDP in singleton gestation, after adjusting for possible confounding factors. Since 1980 the U.S. birth rate for twins has risen significantly, the birth rate for twins in the U.S. was 33.9 per 1,000 live births in 2014. ART is one of the most important causes of twin gestation (30). In 2014, ~ 38.0% of ART-conceived birth were twin birth, and 2.0% were triplets and higher-order birth (31). The number of twin gestations has been steadily growing with the increasing use of ART, so it is very important to access the relationship between HDP of twin-pregnancy women and adverse neonatal outcomes. With regard to twin gestation, there are currently several studies investigating the association of HDP with adverse birth outcomes, but it is rarely performed specifically on the ART population. In 2000, Sibai et al. demonstrated that twin pregnancies complicated by hypertension had a better outcome as compared with both preeclampsia and normotensive twin pregnancies (32). In 2006, a retrospective cohort study by Luo et al. investigated 102,988 twin pregnancies compared to 5,523,797 singletons and found that gestational hypertension in twin pregnancies had overall better neonatal outcomes in terms of rate of preterm delivery, intrauterine growth restriction, neonatal death, and APGAR score (28). Two additional studies also reported that gestational hypertension in twin pregnancies is not detrimental to fetal growth, or even gestational hypertension can be beneficial for fetal survival in twin pregnancies, as compared to normotensive ones (33, 34). A more recent study by Aviram et al. revealed a similar pattern. These findings suggest that the main effect of HDP on the risk of adverse neonatal outcomes is overshadowed by twin gestation (35). In this ART population, it is worth mentioning that, in contrast to ART women with singleton pregnancies, although the absolute rate of adverse neonatal outcomes was higher in twin gestation, regardless of HDP, the risk associated with HDP in twin gestation was lower when compared with the risk associated with HDP in singleton pregnancies.

In addition, accumulating evidence indicated that the risk of HDP varied by different types of ART procedures. According to a US surveillance system for ART-induced births in 2017 by the Centers for Disease Control and Prevention, over 99% of ART procedures involve IVF. For certain IVF procedures, about 75% of them use ICSI technology (36). For example, one study reported more than a twofold increase in the risk of GH/PE in pregnant women treated by ICSI procedures with surgically obtained sperm compared with those treated with IVF procedures (37). A recent meta-analysis also confirmed that IVF/ICSI pregnancies are at higher odds of HDP and preeclampsia than SC, irrespective of the plurality (38). The odds were especially high in frozen embryo transfer and oocyte donation pregnancies. Another meta-analysis by Maheshwari et al. included 26 studies and almost 300 000 deliveries and found that singleton pregnancies conceived through frozen embryos were at lower risk of preterm birth (RR = 0.90; 95% CI 0.84–0.97), low birth weight (RR = 0.72; 95% CI 0.67–0.77), and small for gestational age (RR = 0.61; 95% CI 0.56–0.67) compared to those conceived through fresh embryo transfers, but faced an increased risk of HDP (RR = 1.29; 95% CI 1.07–1.56), large for gestational age (RR = 1.54; 95% CI 1.48–1.61), and high birth weight (RR = 1.85; 95% CI 1.46–2.33) (39). Currently, there is no consensus on this point. Due to a lack of information regarding the types of ART procedures in this study, we could not perform further analysis.

Several possible explanations may account for this observation. This in part may be attributed to twin pregnancies having a higher baseline prevalence of some adverse neonatal outcomes primarily PTB, which may therefore mask the possible added effect of HDP on these outcomes. Physiologically, twin pregnancies have higher cardiac output than singleton pregnancies, further contributing to increased blood pressure. If the mother can withstand the alterations, increased cardiac output is important to accommodate and meet the nutrition demands of the mother and fetus. Previous research demonstrated that low cardiac output in pregnancy was associated with an increased risk of intrauterine growth restriction (40), which is a more serious problem in twins, affecting fetal nutrition and growth. However, maternal high blood pressure increases the risk of preeclampsia, and women with twin pregnancies have a 2–3 times greater risk of developing preeclampsia than the singletons (41). Generally, twin pregnancies include two main types: monochorionic twins and dichorionic twins. Comparatively, monochorionic twins have significantly higher incidences of preterm birth, congenital anomalies, low birth weight, and fetal death than singletons or dizygotic twins, because monochorionic twins are more likely to occur twin transfusion syndrome, selective fetal growth restriction, anemia polycythemia sequence, and cord entanglement and so on (42). In ART pregnancies, twin pregnancies are primarily dizygotic, but fertility treatment can significantly increase the frequency of monozygotic twins from two to 12 times the population incidence of 0.4% (43). However, the rate of preeclampsia is more often in dichorionic than in monochorionic twin pregnancies (44). So HDP in twin pregnancies is very complicated and will need further study. It is known that preeclampsia occurs largely due to an imbalance in prostacyclin and thromboxane A2 (TXA2) (45). Substantial clinical and experimental evidence suggests that low-dose aspirin (60–150 mg), a non-steroidal anti-inflammatory drug, should be initiated in all multifetal pregnancies to reduce the risk of preeclampsia (46, 47). Recently, the American College of Obstetricians and Gynecologists (ACOG) indicated that antihypertensives are not recommended for women with mild gestational hypertension or preeclampsia (systolic blood pressure (SBP) < 160 mmHg or diastolic blood pressure (DBP) <110 mmHg) (8). Moreover, several studies have suggested that lower blood pressure (achieved with the use of antihypertensive medication) vs. higher blood pressure may result in lower birth weight and a heightened risk of SGA newborns (48, 49).

In addition, some investigators have proposed a detection bias in twin pregnancies, who would receive more medical attention, that may be helpful in the appropriate management of HDP as well as many severe maternal or neonatal complications (50). Due to the particularity of the ART population, nevertheless, no obvious difference was found in early prenatal care between singleton and twin groups in our study. These findings suggested that twin gestation is the main cause of adverse neonatal outcomes, and well-controlled preeclampsia is particularly important in twin pregnancies, moderately elevated blood pressure increases blood flow to the placenta, producing a protective effect against adverse birth outcomes for the fetus.

### Strengths and limitations

Our study has several strengths. First, we used a large nationwide population database with detailed information on birth outcomes, mother characteristics, and low levels of missing data of all women who had live births *via* ART to explore the incidence of HDP and its association with a wide range of well-defined adverse neonatal outcomes. Second, this register covers almost the entire region of the United States from both primary and tertiary level institutions, which makes our findings easy to generalize to a wide variety of settings. Third, our data is contemporary, spanning 2015 to 2019 with the credible reference standards for ART pregnancy. Fourth, the maternal and infant clinical characteristics were extracted from the facilities worksheet by hospital staff, particularly eliminating the recall bias.

We acknowledge some limitations. First, this study is limited by its observational design. Although we made every attempt to adjust the known potential confounders including maternal and infant characteristics, undoubtedly the unmeasured confounders possibility exists that might influence birth outcomes. Second, our database lacked detailed clinical information on blood pressure values including systolic and diastolic blood pressure or anti-hypertensive treatments, which makes it impossible to accurately estimate the impact of hypertension or different blood pressure-lowering drugs on neonatal outcomes. Third, we cannot distinguish between ART mothers with different hypertension severity during pregnancy, because gestational hypertension and preeclampsia during pregnancy were combined in this database, and the timing of maternal hypertension diagnosis data was lacking but this is beyond the scope of the current investigation. Fourth, NVSS birth data did not specify the reason for each case of preterm birth, which precludes us from doing analyses for each subtype of preterm birth. Fifth, this database only reported live birth records lacking information about fetal deaths or stillbirths, due to severe HDP can be life-threatening both for the mother and fetus, causing a slight underestimate of the risk of HDP on neonatal outcomes. Moreover, maternal prepregnancy BMI and smoking status before or during pregnancy was based on self-report, it is possible that there was residual confounding. Unfortunately, this database also lacks relevant information about subtypes of ART procedures and twin pregnancies, which limits our further analysis.

## Conclusion

In this large population-based study, ART mothers with HDP were associated with increased risks of adverse neonatal outcomes. However, although twin pregnancies experience much higher rates of HDP, the risk of adverse neonatal outcomes associated with HDP was lower than that of singletons. This information may be useful when counseling ART women with twin pregnancies. Currently, no guidelines for managing HDP specific to multi-fetal gestations, especially for ART-treated mothers, further studies on the evaluation of hypertension in twin pregnancies should consider adding maternal longitudinal cardiovascular parameters changes and indicators of preeclampsia in addition to simple peripheral arterial blood pressure.

## Data availability statement

The original contributions presented in the study are included in the article/supplementary material, further inquiries can be directed to the corresponding authors.

## Ethics statement

Written informed consent was obtained from the individual(s) for the publication of any potentially identifiable images or data included in this article.

## Author contributions

CL, RG, and JH conceived the idea. TL and BX undertook data analysis. TL, CL, YL, and XS wrote draft of the manuscript. All authors were involved in data analysis, drafting the article, or revising it critically for important intellectual content, and approved the final version to be published.

## Funding

This work was supported by the National Natural Science Foundation of China (No. 81903294) and Natural Science Foundation of Guangdong Province (No. 2018030310412).

## Conflict of interest

The authors declare that the research was conducted in the absence of any commercial or financial relationships that could be construed as a potential conflict of interest.

## Publisher's note

All claims expressed in this article are solely those of the authors and do not necessarily represent those of their affiliated organizations, or those of the publisher, the editors and the reviewers. Any product that may be evaluated in this article, or claim that may be made by its manufacturer, is not guaranteed or endorsed by the publisher.
